# Receptor Tyrosine Kinase Ubiquitination and De-Ubiquitination in Signal Transduction and Receptor Trafficking

**DOI:** 10.3390/cells7030022

**Published:** 2018-03-15

**Authors:** William R. Critchley, Caroline Pellet-Many, Benjamin Ringham-Terry, Michael A. Harrison, Ian C. Zachary, Sreenivasan Ponnambalam

**Affiliations:** 1 Endothelial Cell Biology Unit, School of Molecular & Cellular Biology, University of Leeds, Leeds LS2 9JT, UK; w.r.critchley@leeds.ac.uk; 2 Centre for Cardiovascular Biology & Medicine, Rayne Building, University College London, London WC1E 6PT, UK; c.pellet-many@ucl.ac.uk (C.P.-M.); benjamin.ringham-terry@ucl.ac.uk (B.R.-T.); i.zachary@ucl.ac.uk (I.C.Z.); 3 School of Biomedical Sciences, University of Leeds, Leeds LS2 9JT, UK; m.a.harrison@leeds.ac.uk

**Keywords:** receptor tyrosine kinases, ubiquitin, ubiquitin ligases, de-ubiquitinases, signal transduction, trafficking, proteolysis

## Abstract

Receptor tyrosine kinases (RTKs) are membrane-based sensors that enable rapid communication between cells and their environment. Evidence is now emerging that interdependent regulatory mechanisms, such as membrane trafficking, ubiquitination, proteolysis and gene expression, have substantial effects on RTK signal transduction and cellular responses. Different RTKs exhibit both basal and ligand-stimulated ubiquitination, linked to trafficking through different intracellular compartments including the secretory pathway, plasma membrane, endosomes and lysosomes. The ubiquitin ligase superfamily comprising the E1, E2 and E3 enzymes are increasingly implicated in this post-translational modification by adding mono- and polyubiquitin tags to RTKs. Conversely, removal of these ubiquitin tags by proteases called de-ubiquitinases (DUBs) enables RTK recycling for another round of ligand sensing and signal transduction. The endocytosis of basal and activated RTKs from the plasma membrane is closely linked to controlled proteolysis after trafficking and delivery to late endosomes and lysosomes. Proteolytic RTK fragments can also have the capacity to move to compartments such as the nucleus and regulate gene expression. Such mechanistic diversity now provides new opportunities for modulating RTK-regulated cellular responses in health and disease states.

## 1. Introduction

**Figure 1:**
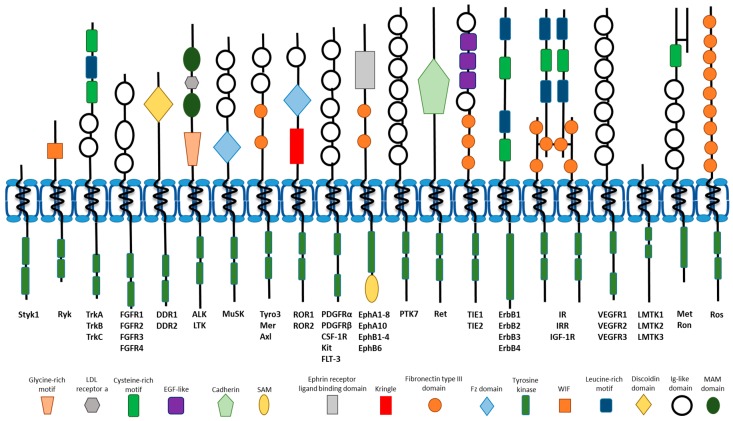
Overview of the domain architecture of the 20 human RTK families. Each RTK contains five fundamental structures: an extracellular ligand-binding domain, a short helical transmembrane region, a juxtamembrane region, cytoplasmic domain with tyrosine kinase activity and a flexible C-terminal tail region. The extracellular ligand-binding domain displays significant variability between families, with notable additions of motifs rich in glycine, cysteine or leucine and immunoglobulin-like domains amongst others. The insulin receptor family differs from the rest due to its pre-assembled multimeric complex.

Receptor tyrosine kinases (RTKs) represent a family of integral membrane proteins exemplified in humans by 58 proteins divided into 20 subfamilies. These include the epidermal growth factor receptor (ErbB), vascular endothelial growth factor receptor (VEGFR), platelet-derived growth factor receptor (PDGFR) and insulin-like receptor (IR) families [1,2,3] (Figure 1). All RTKs exhibit similar domain organisation with an extracellular ligand-binding domain, a single transmembrane region with α-helical propensity followed by a cytoplasmic domain with tyrosine kinase activity. The unique ligand-binding properties of different RTKs are determined by features in the extracellular domain such as fibronectin III-like repeats [4,5], cysteine-rich motifs [6,7], immunoglobulin-like domains [8], leucine-rich and glycine-rich repeats/motifs [9,10] (Figure 1). Interestingly, two branches of the RTK superfamily contain long ~60–100 residue inserts within the TK domain, with potentially significant regulatory roles. Deletion of the kinase insert from VEGFR2 increases tyrosine kinase activity in the activation loop, but also induces a loss of Y1175 phosphorylation and diminished signaling through phospholipase Cγ1 (PLCγ1) [11].

Ligand engagement with the RTK extracellular domain induces conformational changes resulting in RTK activation and downstream signal transduction. For RTKs such as the ErbB, VEGFR or PDGFR families, RTK binding to ligand promotes homo- or heterodimer formation (within each family). In other cases, such as IR, a pre-assembled complex is activated by ligand binding (Figure 1). Interestingly, substantial variability can be observed in the extent of monomeric and pre-formed dimeric RTKs within the same RTK family [12]. One consistent aspect of ligand binding to RTK is transmission of conformational changes through each RTK, resulting in conformational changes in the TK domain [12]. Such changes in TK disposition invariably lead to phosphorylation of neighbouring proteins including other RTKs, signaling adaptors, enzymes and kinases [13]. Activation of the TK domain causes hydrolysis of bound ATP, resulting in the transfer of the γ phosphate group onto the hydroxyl group of a nearby tyrosine side chain. Tyrosine autophosphorylation of the RTK is an early and rate-determining step in controlling the flow of information into different signaling circuits, which in turn modulate cellular responses. Importantly, such modifications are also reversible via the action of protein tyrosine phosphatases (PTPs), which enhances flexibility in controlling signaling output and cell function.

## 2. Signal Transduction

**Figure 2:**
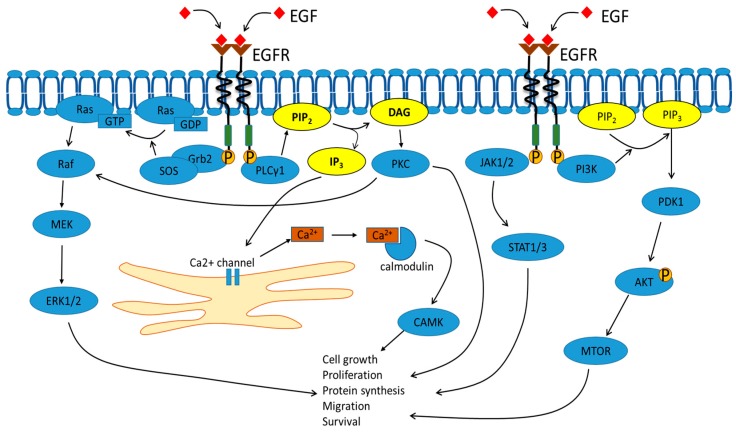
SignalingEpithelial growth factor receptor signal transduction. Stimulation of EGFR with the EGF ligand initiates a series of signaling cascades that significantly alter cellular function. EGFR activation triggers the recruitment of Grb2 and son-of-sevenless to the plasma membrane, promoting Ras-GDP to Ras-GTP transition and activation of the MAPK pathway. Separately, EGFR activation stimulates PLCγ1 activity and PIP_2_ hydrolysis to release diacylglycerol and IP_3_. EGF also mediates downstream signaling through the PI3K/Akt and JAK/STAT pathways to induce cellular changes.

The details of RTK-mediated signal transduction are covered only briefly here, to set the subsequent discussion in context. In-depth analysis of the mechanisms of RTK-mediated signal transduction is featured in this thematic issue, and reviews elsewhere [3,14,15,16]. Briefly, RTKs can undergo activation by both membrane-bound and soluble ligands. RTK activation by ligand induces tyrosine phosphorylation at multiple sites within the cytoplasmic domain, which triggers recruitment of specific adaptors and enzymes to the cytosolic face of the plasma membrane. In the case of EGFR, activation results in the recruitment of Grb2 adaptor which binds to specific phosphotyrosine epitopes, further resulting in binding to the son-of-sevenless (Sos) guanine exchange factor (Figure 2) [17]. Recruitment of Sos to this locality, enables interaction with plasma membrane-localized Ras, a proto-oncoprotein and GTP-hydrolyzing enzyme (GTPase). Sos stimulates Ras-GDP to exchange the bound nucleotide and move into the Ras-GTP state, thus triggering a set of subsequent downstream reactions which is the canonical mitogen-activated protein kinase (MAPK) pathway (Figure 2). Other downstream targets of activated EGFR include PLCγ1, which is again recruited by binding to specific phosphotyrosine epitopes, followed by hydrolysis of plasma membrane-localized phospholipid, phosphatidylinositol-4,5-bisphosphate (PIP_2_). This hydrolysis generates membrane-bound diacylglycerol and soluble inositol-1,4,5-trisphosphate (IP_3_), rapidly diffusible second messengers with potent effects on cell function (Figure 2). Similar pathways can be activated by the different members of the RTK superfamily; however, the location, intensity and duration of such signaling events exhibit unique patterns depending on ligand, RTK and cell-type. In this manner, the complexity of signaling mechanisms has evolved to exquisitely control cell, tissue and animal function.

## 3. Plasma Membrane Endocytosis

An increasingly important concept in RTK functionality is membrane trafficking. The amount of plasma membrane RTK (and thus free RTK available for exogenous ligand sensing) is substantially influenced by the rate of newly synthesized RTK arriving from the secretory pathway, balanced by the rate of internalization (endocytosis) at the plasma membrane. Typically, a newly synthesized RTK polypeptide is made as a Type I integral membrane protein which undergoes translocation into the endoplasmic reticulum (ER), signal peptide cleavage, usually followed by initial stages of N- and O-linked glycosylation [18]. Interestingly, a recent study suggests that activation of EGFR causes increased gene expression of components of the COPII machinery that mediates ER-to-Golgi trafficking [19], suggesting that modulating RTK secretion could have major impacts on animal physiology.

Subsequent exit from the ER and delivery to the Golgi complex is marked by extensive glycosylation within the different Golgi subcompartments (*cis*, *medial*, *trans*). Exit from the most distal Golgi subcompartment *trans*-Golgi network (TGN) via the constitutive secretory pathway results in delivery to the plasma membrane. Notably, some RTKs display steady-state or distinct patterns of Golgi localization, but the mechanisms underlying such distribution are unclear. For example, c-Met and EGFR are both routed through the Golgi complex to the basolateral plasma membrane in polarized epithelial cells [20,21]. VEGFR1 is reported to be largely present within a Golgi-like subcompartment in endothelial cells but translocates to the cell surface via a cytosolic calcium ion-dependent membrane trafficking route [22]. VEGFR2 shows significant steady-state Golgi localization in a SNARE- and microtubule-dependent manner with functional consequences for signal transduction and new blood vessel sprouting i.e., angiogenesis [23,24,25]. Both tropomysin receptor kinase A (TrkA) and EGFR trafficking through the Golgi complex is SNARE-dependent with functional consequences for signal transduction and functionality [26,27]. Interestingly, one study reports that diffusible reactive oxygen species (ROS) can promote ligand-independent phosphorylation of VEGFR2 within the Golgi complex, resulting in proteolysis and loss of transport to the cell surface [28]. A recent study also suggests that ubiquitination programs the marked loss of maturation of newly synthesized VEGFR2, associated with a reduction in cell surface expression, loss of signaling and diminished levels of angiogenesis [29].

**Figure 3:**
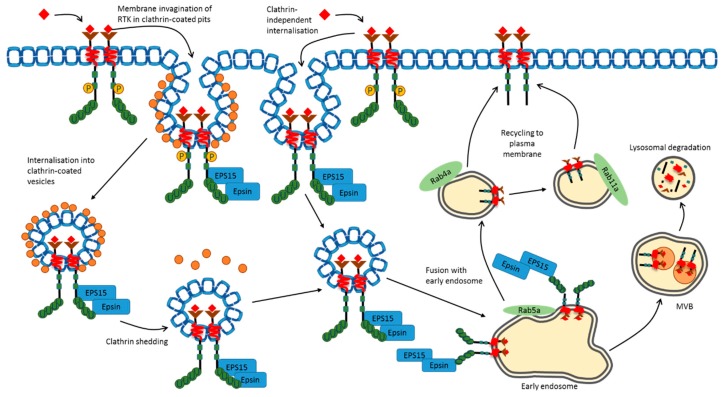
Ubiquitin-mediated internalization of the RTK. RTKs are internalized via both clathrin-dependent (CDE) and clathrin-independent (CIE) pathways following ubiquitination. Essential endocytic components including epsin and eps15 are recruited to the ubiquitinated RTK. Epsin contains two ubiquitin-binding motifs allowing its recognition of the internalization signal, enabling uptake via both CDE and CIE. Both pathways converge in endosomes following the shedding of clathrin (orange circles) from the vesicular surface. Once within the endocytic vesicle, the RTK is delivered by fusion with the Rab5a-expressing early endosome. At this point, RTK fate is decided by either direction to Rab4a-expressing endosomes for recycling or to multivesicular bodies for lysosomal degradation.

Complex mechanisms exist to direct both basal (non-activated, resting, steady-state) and activated RTK into transport pathways that deliver these membrane-bound enzymes to the endosome-lysosome network. Endocytosis of RTKs is well-documented, with clathrin-dependent endocytosis (CDE) of both basal and activated RTKs (Figure 3). STAT3 signaling downstream of PDGFRβ is reliant upon its internalization, which can be mediated by CDE [30]. Regulation of EGFR [31,32,33] and VEGFR2 [34,35,36,37] internalization by CDE impacts on the intensity and duration of downstream signaling. Furthermore, EGF stimulation of EGFR induces PLCγ1 and calcium-dependent signaling responsible for the selective internalization of the receptor by CDE [38]. The vascular regulator Tie2 is linked to internalization via CDE on endothelial cells [39]. There is also evidence that EGFR activation leads to phosphorylation of components of the CDE machinery, thus modulating the rate of internalization [40,41]. Signaling adaptors such as Grb2, which are recruited to EGFR, further modulate CDE [42]. Recent evidence suggests that the adaptor intersectin-s is an important factor in CDE-dependent internalization of EGFR and downstream signaling, and represents a key regulator of recycling [43]. Plasma membrane microdomains containing either lipid rafts or CDE machinery can specifically recruit EGFR [44,45]. It has also been postulated that EGFR undergoes differential post-translational modifications and proteolytic fates depending on its association with CDE or lipid rafts [46]. One likelihood is that CDE modulates RTK-linked early trafficking events and signal transduction but does not regulate proteolysis in later compartments such as late endosomes and lysosomes [32].

However, clathrin-independent endocytosis (CIE) has also been documented for RTKs such as EGFR, PDGFRβ and VEGFR2, with roles for lipid rafts, caveolae and macropinocytosis postulated to direct the internalization and delivery to endosomes [30,47,48]. EGFR association with caveolae and lipid rafts is dependent on a cysteine-rich region of the extracellular domain close to the transmembrane region [49], suggesting involvement of accessory membrane proteins or co-receptors. VEGFR2 association with CIE-dependent pathways such as lipid rafts and caveolae [50] is balanced by internalization via CDE [22,34]. Another RTK called ROR is implicated in functioning as a scaffold to bind cavin-1 and caveolin-1, structural components of caveolae [51]. RTK ligands, such as insulin, EGF, and VEGF-A, stimulate c-Src recruitment, activation and tyrosine phosphorylation of caveolin-1 [52,53,54]. An emerging theme from multiple studies is that RTK recruitment to different microdomains is dynamic, and potentially activation-dependent. RTK association with either CDE or CIE leads to modulation of internalization, trafficking and proteolysis, which has major consequences for downstream signal transduction events. It is likely that RTK activation could promote recruitment of downstream effectors (e.g., c-Src) or directly stimulate phosphorylation of structural components of CDE and/or CIE pathways that promote or inhibit inclusion into microdomains. Such microdomain clusters could mediate stable plasma membrane association or specify internalization and delivery to endosomes.

Increasingly, it has been postulated that many activated RTKs including EGFR and VEGFR2 trigger important signaling events in endosomes, including the canonical mitogen activated protein kinase (MAPK) and phosphoinositide 3-kinase (PI3K) signaling pathways [31,55,56]. Enhanced EGFR signaling is observed when trafficking from early to late endosomes is slowed, indicating the importance of the endosomal compartment as a signaling station [57]. Another RTK, c-Met, upon activation, requires endocytosis and delivery to endosomes to signal through the STAT3 pathway [58]. The capacity of an activated RTK to signal from a variety of endosomal compartments appears likely. Indeed, c-Met al.so displays differential signaling patterns depending upon its localization within peripheral or perinuclear endosomes [59]. Activated PDGFR can signal to the PI3K and PLCγ1 pathways from endosomes, leading to physiological responses [60]. Notably, blocking VEGFR2 endocytosis at the cell surface diminishes signal transduction due to reduced endosome occupancy [35]. The extended residency of VEGFR2 within sorting endosomes is associated with a loss of signaling, demonstrating the complexity in endosomal regulation of signal transduction downstream of the RTK [61]. Further RTK trafficking to multivescular bodies and lysosomes is dependent on ubiquitin chain recognition closely linked to proteolysis and signal cessation.

## 4. Ubiquitin Modification and Ubiquitin Ligases

An important aspect of biochemical regulation of eukaryote function is the use of small, ubiquitin-related protein tags to target proteins for destruction to enable down-regulation and recycling of protein-based components. We now know that such ubiquitin-like tags can cause three different outcomes: target proteins for proteolysis, target proteins to specific locations within a cell and/or modulate biochemical or enzymatic activity. In the case of RTKs, there are increasing examples of RTK modification by the attachment of monomer or polymeric forms of ubiquitin to lysine residues at one or more sites within their cytoplasmic domains [62,63,64]. Recently, it has been noted that basal (non-activated) VEGFR2 undergoes ubiquitination, which consequently has substantial effects of ligand-stimulated signal transduction and cellular responses [65]. The attachment of ubiquitin to RTKs such as the ErbB and VEGFRs [65,66,67] appears to mediate inclusion into CDE and efficient delivery through the endosome-lysosome system for proteolysis. In this way, RTK degradation enables cessation of signal transduction.

**Figure 4:**
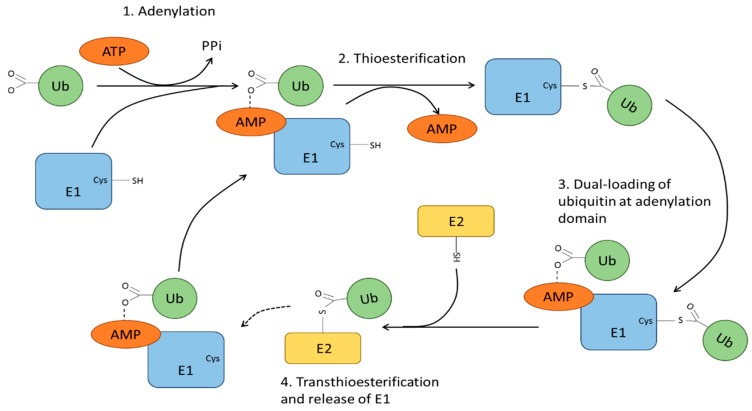
Ubiquitin activation and conjugating by E1 and E2 enzymes. The E1 enzyme binds to either ubiquitin or ubiquitin-like modifier and Mg-ATP, resulting in ATP hydrolysis. This allows the adenylation of the ubiquitin/ubiquitin-like modifier and release of pyrophosphate. This is followed by the formation of a thioester bond between a cysteine residue within the E1 and the C-terminal glycine of the ubiquitin molecule accompanied by release of AMP. The E1 subsequently undergoes a second round of ubiquitin loading at the adenylation site prior to interaction with the E2. The transfer of the ubiquitin from E1 to a cysteine residue on the E2 occurs via transthioesterification, allowing the release of the E1.

Ubiquitin is a highly conserved 76 amino acid polypeptide encoded by 4 human genes, i.e., UBB, UBC, UBA52 and RPS27A [68,69]. UBB and UBC represent polymeric concatemers with three and nine consecutive repeats respectively. UBA52 and RPS27A encode for monoubiquitin fused to the ribosomal proteins L40 and S27A. Both ubiquitin forms are processed to release free ubiquitin monomers to maintain an intracellular ‘free’ ubiquitin pool. This steady-state ubiquitin pool is utilized by 3 enzyme families, termed E1, E2 and E3 ubiquitin ligases. The E1 (ubiquitin activating enzymes), E2 (ubiquitin conjugating enzymes) and E3 (ubiquitin ligases), work in concert to orchestrate addition of ubiquitin to the ε-amino side chain on specific lysine residues within a protein substrate or target. The E1 family has 10 members, with only 2 that are specific to ubiquitin chain addition. These 2 enzymes, UBA1 [70] and UBA6 [71], are structurally similar and are able to initiate the first step of the ubiquitination cascade (Figure 4). The remaining 8 E1 family members are UBA2 [72], UBA3 [73], UBA4/MOCS3 [74], UBA5 [75], UBA7 [76], NAE1 [77], SAE1 [72] and ATG7 [78], which are responsible for activation of ubiquitin-like tags for addition to target substrates. These protein tags include small ubiquitin-related modifier (SUMO), neural precursor cell expressed, developmentally downregulated 8 (NEDD8), and ubiquitin-fold modifier (Ufm). Although sumoylation, neddylation and ufmylation use different biochemical pathways from ubiquitination, the essential features of the cellular machinery involved is mechanistically similar, and thus require interactions with downstream E2 and E3 enzymes.

E1s bind their cognate ubiquitin or ubiquitin-like modifier and Mg-ATP linked to subsequent ATP hydrolysis to AMP and pyrophosphate, enabling thioester bond formation between a critical cysteine side chain and the carboxyl-terminal glycine in ubiquitin (Figure 4). This E1-Ub subsequently undergoes a second round of ubiquitin linkage and the dual-loaded E1-Ub species is now competent for interaction with E2 enzymes (Figure 4). Recent evidence demonstrates that UBA5 differs from the other E1 enzymes in its requirement for trans-binding of the UFM1 to the UBA5 homodimer [79]. UFM1 binding stabilises the UBA5 homodimer, enhancing ATP binding [80]. Recruitment of an appropriate E2 to E1-Ub is a critical event in the progression of the ubiquitination cycle, and handover of these ‘charged’ ubiquitin substrate onto E2 enzymes (Figure 4).

This E2 ubiquitin ligase family encoded by ~45 genes is defined by a conserved ~150 residue ubiquitin-conjugating (UBC) domain, exemplified by a cysteine residue needed for the transfer of the charged ubiquitin (from E1) via a thioester bond with E2, and a histidine-proline-asparagine (HPN) triad that regulates E2 activity. The histidine residue represents a critical structural element, providing functional stability within the E2 enzyme [81]. The asparagine residue within the HPN triad catalyses the formation of an isopeptide bond between the ‘charged’ ubiquitin and the target substrate. This occurs by stabilization of the oxyanion intermediate which regulates interaction of the E2-ubiquitin thioester with target lysine side chain on the target substrate [81,82]. However, this step also requires E2 interaction with a corresponding or specific E3 ubiquitin ligase. The E3 protein can function as an adaptor but can also possess catalytic properties. Such features enable the target substrate to achieve close molecular proximity to the charged E2-Ub species. Although the E2 family already provides diversity, the E3 family is even more diverse with >700 E3s encoded within the human genome; the exact number of E3 family members uncertain at present. Substrate specificity is dependent on the E2:E3 complex, with a single E2 able to interact with multiple E3s.

**Figure 5:**
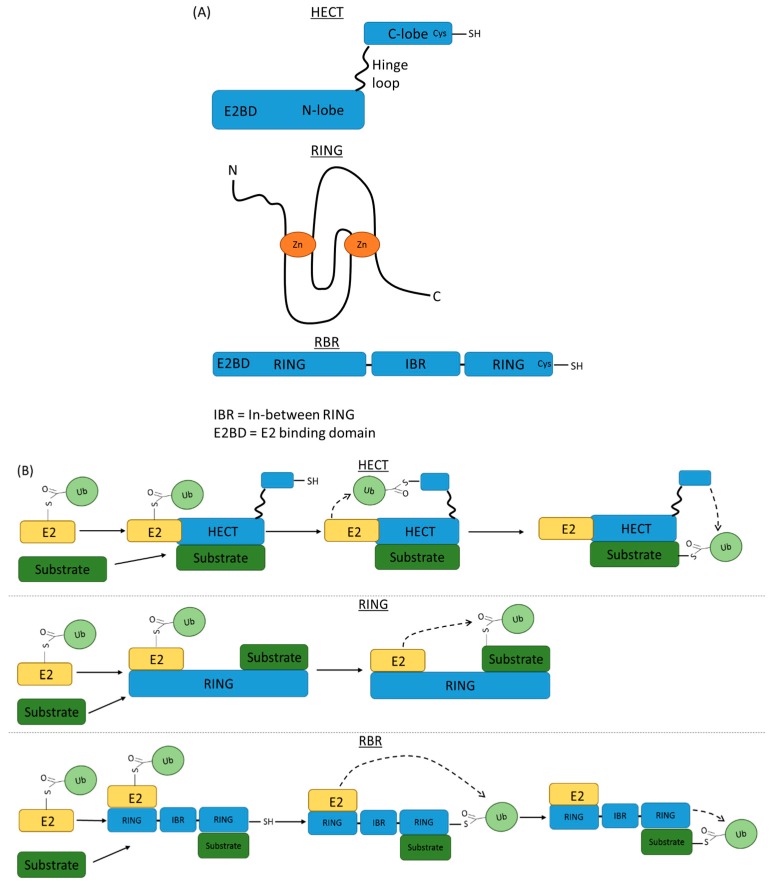
HECT, RING and RING between RING E3 families differ in their mechanism of ubiquitin transfer to substrates. (**A**) The major E3 families HECT, RING and RING between RING display distinct structures that determine their mode of ubiquitin transfer. HECT E3s have a hinge loop connecting their N-lobe, which contains the E2 binding site, with their C-lobe containing the cysteine residue critical for ubiquitin linkage. RING E3s typically display conserved cysteine and histidine residues within their core structure that stabilise the presence of central zinc ions. The loop structures surrounding the zinc ions represent the conserved E2 binding site. RING between RING E3s are complex multidomain proteins containing two RING domains separated by an ‘in-between RING’ domain. The N-terminal RING contains the E2 binding site and the C-terminal RING contains the catalytic cysteine residue. (**B**) The mechanism of ubiquitin transfer differs between the E3 families. HECT E3s bind to the substrate and ubiquitin-charged E2, which induces a change in the orientation of the hinge loop to move the C-lobe and catalytic cysteine into closer proximity to the ubiquitin. Ubiquitin is then transferred from the E2 to the C-lobe cysteine, enabling a return to the original lobe orientation and transfer of the ubiquitin from the HECT E3 to a lysine residue on the substrate. RING E3s differ significantly from this as their role is more of a scaffold or adaptor that binds both E2 and substrate. This brings both ubiquitin and substrate into close proximity and enables the direct transfer of ubiquitin from the E2 to a lysine residue on the substrate. Mechanistically, the RBR E3 acts in a more similar manner to their HECT counterparts. The RBR binds the E2-ubiquitin to its N-terminal RING and binds the substrate with its C-terminal RING. An intermediate thioester bond is formed between the ubiquitin and the C-terminal RING. The ubiquitin molecule is subsequently transferred to a lysine residue on the target substrate that is bound by the C-terminal E3 RING.

E3 family members can be subdivided into three groups, including the homologous to the E6AP carboxyl terminus (HECT), really interesting new gene (RING) and RING between RING (RBR) groups [83] (Figure 5A). The HECT group, which was the first E3 class to be described, has 28 members [84,85]. The HECTs include a C-terminal domain containing a catalytic cysteine residue and an N-terminal domain with an E2 binding motif [86] (Figure 5A). RING E3s comprise the largest group, with at least 600 members, including the structurally similar U-box proteins [87], and contain conserved cysteine and histidine residues in the core that bind zinc ions. The RBR group members have two RING domains separated by an in-between RING domain [83].

Mechanistically, HECT enzymes form an intermediate thioester bond with the carboxyl-terminus glycine residue of the charged ubiquitin (attached to E2) prior to transfer to the target substrate [88] (Figure 5B). In contrast, RING members appear to facilitate direct transfer of the charged ubiquitin from E2 to the lysine side chain on the target substrate [89]. The formation of the E2-Ub:E3 RING complex could facilitate a change in the ‘closed’ conformation in the E2-Ub intermediate, thereby priming the thioester linkage for nucleophilic attack on the closely aligned lysine(s) within the target substrate [90] (Figure 5B). RBRs display hybrid features of both RING and HECT groups. Typically, an RBR has E2 binding capacity through the N-terminal RING1 domain and a catalytic cysteine residue within the C-terminal RING2 domain allows transfer of charged ubiquitin from the E2 intermediate to the RBR, before being conjugated to a specific lysine side chain on the target substrate (Figure 5B).

Multiple types of ubiquitination chains attached to target substrates are dependent on the 7 lysine residues (K6, K11, K27, K29, K33, K48, K63) and free primary amino terminus in the N-terminal methionine (M1) within the ubiquitin polypeptide [91]. The nature of polyubiquitin chains is highly variable, with the potential for several branches of ubiquitin chains to form at different lysine residues on the same substrate-bound ubiquitin, and the possibility of mixed-chain species with other ubiquitin-like proteins also being found within the same chain alongside ubiquitin [92]. This corresponds to a significant number of potential effects on cellular function, although limited data is available for atypical chain linkages. One such function of ubiquitination that has been explored in some depth is in the DNA damage response pathway [93,94,95,96,97,98]. K6 and K33 polyubiquitin chains in particular have been linked with the DNA damage response [99], alongside K27 chain modification of chromatin [100], although further research is necessary to confirm these roles. Branched K11 polyubiquitin chains demonstrate more efficient trafficking of the tagged substrate to the proteasome than other types of ubiquitin linkage, suggesting a major role in protein degradation [101]. K29 chains may play an inhibitory role in signaling through the Wnt/β-catenin pathway [102]. K48 chains are the most commonly observed linkage type and provide a strong signal for protein degradation [103]. K63 chains are also associated with protein degradation [104], as well as cell signaling pathways [105]. Mixed K48-K63 branched species may also play a major role in NF-κB signaling [106], but also direct tagged proteins for degradation [107]. M1 linear ubiquitin chains are strongly associated with innate immune signaling [108] and have a well-documented role in TNF receptor function [109].

## 5. Plasma Membrane RTK Ubiquitination and Sorting

Integral membrane proteins such as RTKs appear to undergo both monoubiquitination, multi-monoubiquitination and polyubiquitination (also at single and multiple sites) on the protein backbone via attachment to lysine side chains. The overall effects on the RTK may differ substantially depending upon the exact location of the sites of attachment, number of ubiquitin linkages, and exact nature of ubiquitin chains. Ubiquitination of cell surface receptors is generally thought to target these proteins for destruction in the late endosome and lysosome; however, the exact mechanism(s) remain elusive. Currently, the view is ubiquitinated RTKs exhibit increased proteolysis, resulting in cessation of signaling compared to non-ubiquitinated RTKs [110]. RTK ubiquitination is thought to promote endocytosis via CDE and/or CIE [111,112]. Modifying RTK by attachment of different ubiquitin tags is a complex strategy to control receptor availability at the plasma membrane and dictate trafficking before recycling or proteolysis. It is well-established that for RTKs such as EGFR, ErbB2, MuSK [113], TrkA [114] and VEGFR2 [6,34], ligand activation precedes ubiquitination and is linked to increased degradation in the endosome-lysosome network. Activation of TrkA by nerve growth factor occurs in response to ligand stimulation and can be mediated by both multimonoubiquitination [114] and polyubiquitination [115,116]. VEGFR2 ubiquitination is associated with downregulated cell surface expression and blockade of receptor ubiquitination significantly impairs endocytosis resulting in increased cell surface levels [29]. Similarly, monoubiquitination of activated PDGFR or EGFR is sufficient to stimulate endocytosis, reduce cell surface levels and availability for exogenous ligand binding [117]. Activated EGFR displaying K63-linked polyubiquitin chains are subjected to increased proteolysis [118]. Many studies suggest that RTK ubiquitination can be a signal to promote endocytosis resulting in down-regulation of cell surface levels, and this is irrespective of the type of ubiquitin chain attachment.

Transport from the cell surface necessitates the involvement of a group of proteins known as the endosomal sorting complexes required for transport (ESCRT). ESCRT recognition of the type of ubiquitin attachment conjugated to RTKs is dependent on endocytic machinery such as the proteins epsin and Eps15, both of which contain ubiquitin-interacting motifs (UIMs) [119,120,121]. ESCRTs play key roles in the recognition and trafficking of ubiquitinated EGFR. However, ESCRT-0 and ESCRT-II complexes are dispensable for EGFR recycling back to the plasma membrane from endosomes [122]. The Gα_s_ protein has recently been demonstrated to bind to the ubiquitinated Hrs component of ESCRT-0, and depletion of Gα_s_ delays EGFR degradation by impeding trafficking into lysosomes [123]. ESCRT-III proteins also have a demonstrable role in regulating EGFR trafficking, as evidenced in recent studies with depletion of ESCRT-III regulatory proteins CC2D1A/CC2D1B, which curtailed EGFR signaling and accelerated EGFR degradation [124]. Studies assessing the internalisation of c-Met and EGFR have both demonstrated requirement for a complex comprising the proto-oncoprotein and E3 ligase (Cbl), CIN85 and endophilin [125,126]. This early endocytosis-associated complex acts to encapsulate the receptor into early invaginations at the plasma membrane and promote inclusion into endocytic vesicles. Such vesicles then fuse with early endosomes, a key compartment for RTK signal transduction and sorting. Inhibition of Cbl association with CIN85 by the GTP-binding protein SEPT9 impairs EGFR ubiquitination and degradation [127].

## 6. RTK Ubiquitination, Recycling and Proteolysis

Plasma membrane RTK down-regulation as a consequence of ubiquitination was conventionally viewed to program cessation of signal transduction after reaching multivescular bodies (MVBs) or late endosomes, followed by degradation in lysosomes. This would provide a means for RTK signal attenuation or cessation. Whilst sorting of EGFR into MVBs before proceeding to lysosomes for proteolysis is well-established [128,129], other studies also suggest that EGFR, TrkA and VEGFR2 undergo proteolysis by a proteasome-regulated pathway [9,116,130].

RTKs can be recycled from endosome-to-plasma membrane to maintain sensitivity to exogenous ligand, although it is unclear whether signaling continues during recycling [131,132,133,134,135,136,137]. Mutation of specific lysines within the FGFR1 cytoplasmic domain suggest that the efficiency of recycling is increased and targeting for degradation is diminished, facilitating increased signal transduction [138]. The EGFR model continues to provide new insights and its ability to bind different ligands such as EGF and transforming growth factor-α indicate programming for different signaling and proteolysis outcomes. EGFR:EGF activation stimulates degradation whereas EGFR:TGF-α stimulates recycling to the cell surface [139]. One possibility is that within the lower pH in endosomes, EGF-EGFR remains stably bound but TGF-α rapidly dissociates, allowing efficient EGFR recycling from endosome-to-plasma membrane. Other EGFR ligands suggest the capacity to program differential post-translational modifications which affect EGFR fate through trafficking, recycling or proteolysis [140,141,142]. The family of E1, E2 and E3 ubiquitin ligases is thus critical in determining how RTK fate and signal transduction properties are regulated in this context.

Although RTK ubiquitination is an increasingly important aspect of cellular regulation, there is a dearth of information on E1, E2 and E3 enzymes in this process. Of note, a recent study identified the E1 family member, UBA1, in regulating the ubiquitination of basal (non-activated) VEGFR2 but surprisingly, not for ligand-activated VEGFR2 [65]. This study puts forward the idea that at least 2 different mechanisms of ubiquitination exist, one to modify basal RTKs, and another pathway which targets activated RTKs [65]. It is likely that other RTKs may also exhibit such regulation. The roles for the other 9 E1 family members in RTK regulation remain to be described. There is also increasing evidence for SUMOylation of RTKs, linked to nuclear translocation of proteolytic fragment(s) of the processed RTK e.g., insulin-like growth factor 1 receptor (IGF-1R) [143] and ErbB4 [144], suggesting roles for E1 members which regulate SUMOylation. The identification and mechanistic role of E2 ubiquitin ligases in RTK modification remain to be elucidated. Studies on IGF-1R SUMOylation and nuclear translocation found that such effects were regulated by the E2 family member and SUMO-conjugating enzyme UBE2I [145].

## 7. RTK Targeting by E3 Ubiquitin Ligases

The best-studied aspect of RTK ubiquitination is interaction with E3 ubiquitin ligases that affect signal transduction, trafficking and proteolysis. Many RTKs, including EGFR, PDGFRs, TrkA, Ret, EphRs, FGFRs and VEGFRs, are targeted for ubiquitination by the E3 enzyme Cbl, a proto-oncoprotein that modulates signal transduction, membrane trafficking and proteolysis [146,147,148,149,150,151,152,153,154,155,156,157,158]. Cbl also regulates movement of EGFR out of the early endosomes and facilitates ubiquitination, leading to its proteolysis [147]. Cbl deficiency or disruption of its ability to bind target substrates causes increased PDGFRα and FGFR2 cell surface levels concomitant with reduced ubiquitination [149]. Both c-Cbl and related Cbl-b can also modulate PDGFRβ status with subsequent effects on endocytosis and signal transduction [150]. Other E3 enzymes have been implicated in EGFR modification, including UBE4B [159] and Cullin-3/speckle-type POZ protein-like, SPOPL [160]. Whereas UBE4B is recruited to endosomes and may directly regulate EGFR ubiquitination, Cullin-3/SPOPL targets Eps substrate 15 (Eps15) on endosomes [160], which regulates EGFR recycling [131]. MuSK ubiquitination has been reported to be effected by the E3 ligase PDZ domain-containing ring finger 3, although its role in the endocytosis of other RTKs has not been established [113]. Intriguingly, a recent report suggests that the E3 family member, RNF121, binds to newly synthesized VEGFR2 and impairs its maturation and transport to the plasma membrane [29].

Insulin receptor (IR) cell surface expression is regulated by ubiquitination associated with the E3 ligase TRIM72, which targets both the receptor and its associated scaffold protein insulin receptor substrate 1 (IRS1) for degradation [161]. More recently, the E3 ligase MARCH1 was also shown to diminish IR surface expression through the induction of ubiquitin-mediated degradation [162]. In a similar manner to the effect of UBA1 on basal but not ligand-activated VEGFR2, MARCH1 impacts upon only basal IR surface expression. This may represent a method of fine-tuning the level of receptor available for engagement by ligand. IGF-1R modification is linked to a number of E3 enzymes including c-Cbl [163], Nedd4 [164] and Mdm2 [165]. Interestingly, ubiquitination of IGF-1R by Mdm2 occurs under low IGF-1 conditions but switches to dependence on c-Cbl at high IGF-1 levels. Both c-Cbl and Mdm2 catalyze IGF-1R ubiquitination at different sites; furthermore, Mdm2 specifies IGF-1R modification by attachment of K63-linked polyubiquitin chains, whereas c-Cbl mediates K48-linked polyubiquitin chain attachment [163]. Such biochemical modifications are reflected in differential endocytic routing of IGF-1R with Mdm2 regulation associated with CDE, whereas Cbl is associated with CIE. Importantly, Mdm2 regulation of IGF-1R is associated with proteasome-mediated degradation [165], which further supports a role for K63-linked polyubiquitin chains in delivering substrates to the 26S proteasome [118]. IGF-1R modification by another E3 enzyme, Nedd4, in association with Grb10 leads to endocytosis, lysosomal degradation and eventual down-regulation of signal transduction events [166]. Here, the Nedd4/Grb10 complex is associated with multi-monoubiquitination of IGF-1R. There is also some evidence that Nedd4, Grb10 and ESCRT-0 have complex regulatory roles in VEGFR2 modification, trafficking and proteolysis [167,168].

## 8. RTK De-Ubiquitination

De-ubiquitinases (DUBs) represent a class of proteases that cleave ubiquitin or ubiquitin-like modifiers from attachment to substrate proteins. The DUB superfamily comprises ~120 genes that can be further classified into seven groups, based on the primary sequence of the catalytic domain. The largest DUB group comprises the ubiquitin-specific processing proteases (USPs), and is made up of 56 members. These proteins contain a conserved core USP domain, a Cys-X-X-Cys zinc-binding motif and the catalytic cysteine residue, which classifies this as a cysteine protease [169]. Other DUB cysteine proteases include the ovarian tumour (OTU) group, which contains 16 active gene products [170] and the inactive FAM105a member. The group of ubiquitin C-terminal hydrolase (UCH) cysteine proteases comprises four members with little sequence conservation and catalytic domains ranging from 223 to 729 amino acids. The group of Machado-Josephin domain (MJD) DUBs has 4 members that contain a conserved catalytic Josephin domain [171].

A more recently identified and unique DUB group is the motif interacting with novel ubiquitin-containing novel deubiquitinase family (MINDY) with at least four members [172]. Structural studies show that MINDY enzymes have conserved cysteine and proximal histidine residues that enable nucleophilic attack on the ubiquitin and ubiquitin-like substrates, although the third member of the canonical catalytic triad remains to be identified [172]. The DUB group of the monocyte chemotactic protein-inducible proteins (MCPIPs) has at least four members [173] linked to immune regulation. These zinc-finger containing enzymes reduce cellular ubiquitination but are inhibited by the cysteine protease inhibitor N-ethylmaleiimide [174]. The group of Jab1/Pab1/MPN domain-associated metalloisopeptidase (JAMM) DUBs has at least 12 members, which are zinc metalloproteases, and thus distinct from the DUB cysteine proteases. There is also a group of SUMO-specific proteases with at least 8 members, including ubiquitin-specific protease-like 1 (USPL1), SUMO/sentrin specific proteases (SENP) or desumoylating isopeptidases (DESI).

The removal of ubiquitin and ubiquitin-like modifications on target substrates by DUBs enables re-use of cellular factors, obviating the need for new protein synthesis. Control of DUB activity is thus an essential feature of cellular regulation, enabling the re-activation or re-use of proteins. De novo synthesis of ubiquitin produces either polymeric forms or monoubiquitin precursors fused to ribosomal proteins, rather than free ubiquitin. Both precursor forms require processing by DUBs to release single ubiquitin units within the cell [175]. Recent evidence suggests that monoubiquitin release from its precursors is mediated predominantly by five main DUBs: ubiquitin thioesterase otulin, UCHL3, USP5, USP7 and USP9X [176], although each has multiple substrates that can be targeted for deubiquitination to maintain the free ubiquitin pool. Removal of ubiquitin from a target protein from substrates such as ubiquitinated RTKs allows a new level of regulation of signal transduction that further impacts on cellular responses associated with RTK function. DUBs have specificity for polyubiquitin chain cleavage [177,178,179]. Both recycling and degradation pathways are regulated by ubiquitination [128], with polyubiquitin chain addition to target substrates strongly linked to proteolysis [180]. DUB activity thus controls RTK expression and provides a mechanism by which the activated and ubiquitinated RTK can be redirected away from the degradation pathway towards a return to the plasma membrane. As DUB activity can significantly affect cellular processes that could lead to a variety of pathologies, these enzymes have received attention as therapeutic targets [181].

A reverse genetics screen of human DUBs involved in EGFR regulation has identified an important role for USP18 [182]. Loss of USP18 inhibits cell proliferation and increases apoptosis [183]. However, USP18 regulates EGFR mRNA translation of the EGFR rather than trafficking and proteolysis [183]. The JAMM member, AMSH, is implicated in regulating EGFR de-ubiquitination on endosomes [184]; furthermore, AMSH interacts with components of the ESCRT-III complex [185]. A more recent siRNA screen has identified a number of DUBs that significantly modulate EGFR levels and degradation kinetics [186]. USP9X was a key factor identified from this screen, although it does not directly target EGFR itself, but instead cleaves ubiquitin from endocytic accessory proteins including Eps15. Deubiquitination of TrkB by the DUB UCHL1 has been reported, which maintains expression of the receptor on the cell surface [187]. Loss of UCHL1 diminished TrkB expression at the plasma membrane and programmed its trafficking to the degradation pathway, leading to abrogated RTK signaling.

Another DUB enzyme, USP8, functions similarly to target Eps15 [188]. Transgenic mice with conditional USP8 inactivation develop fatal liver failure associated with the marked downregulation of several RTKs including EGFR, c-Met and ErbB3 [189]. USP8 has been demonstrated to be important in the regulation of c-Met levels indirectly through regulation of leucine-rich repeats and immunoglobulin-like domains 1 protein, LRIG1 [190]. Ubiquitinated LRIG1 recruits c-Met to lysosomes for degradation; reduction in LRIG1 ubiquitination by USP8 depletion or inactivation thus enables c-Met levels and functionality to be maintained. USP8 can form complexes with the ESCRT-0 subunit STAM on endosomes, stimulating de-ubiquitination of EGFR on endosomes, and promoting EGFR endosome-to-plasma membrane recycling [191]. Impairment of USP8 activity has significant implications, with USP8 mutations that increase catalytic activity promoting de-ubiquitination of EGFR and increased sensitivity to EGF and development of Cushing’s disease [192]. A recent study has implicated USP8 requirement in VEGFR2 de-ubiquitination and recycling [193]. Although other DUBs are likely to have important roles in RTK de-ubiquitination, the majority of recent studies have identified USP8 as a major therapeutic target in many disease states [194,195].

## 9. Conclusions

The RTKs represent a diverse group of families that propagate signals derived from binding their cognate ligands via transduction along a milieu of intracellular signaling pathways. These circuits enable the sensing of changes in the extracellular environment (e.g., growth factor levels) and the translating of such information into appropriate cellular responses. RTKs share common regulatory mechanisms that control cellular sensitivity to diverse soluble and membrane-bound ligands. Whilst ubiquitination has a well-documented role in RTK endocytosis, and thus influences cell surface RTK levels, a deeper understanding of the underlying machinery that controls RTK post-translational modification and distribution is needed. Indeed, it is evident that RTK ubiquitination does not simply act as a simple signal for lysosomal degradation, but modulates the balance between the rate of RTK synthesis, recycling and degradation in different intracellular compartments. Moreover, attachment of different ubiquitin tags to a specific RTK opens up the possibility of modulating different signal transduction pathways which are influenced by RTK residence within a specific location. Ubiquitin-modifying enzymes comprising E1, E2, E3 and DUB families may be further positioned at these different locations to influence RTK modification, turnover and distribution. Such effects would invariably impact on the steady-state or basal RTK levels at the plasma membrane, thus dictating RTK availability for sensing exogenous circulating ligand(s). Such ubiquitin-modifying enzymes are increasingly important therapeutic targets for targeting RTK function in diverse disease states including cancer, heart disease and diabetes.

## References

[1] Robinson D.R., Wu Y.M., Lin S.F. (2000). The protein tyrosine kinase family of the human genome. Oncogene.

[2] Blume-Jensen P., Hunter T. (2001). Oncogenic kinase signaling. Nature.

[3] Lemmon M.A., Schlessinger J. (2010). Cell signaling by receptor tyrosine kinases. Cell.

[4] Marino-Buslje C., Mizuguchi K., Siddle K., Blundell T.L. (1998). A third fibronectin type iii domain in the extracellular region of the insulin receptor family. FEBS Lett..

[5] Tuzi N.L., Gullick W.J. (1994). Eph, the largest known family of putative growth factor receptors. Br. J. Cancer.

[6] Garrett T.P., McKern N.M., Lou M., Frenkel M.J., Bentley J.D., Lovrecz G.O., Elleman T.C., Cosgrove L.J., Ward C.W. (1998). Crystal structure of the first three domains of the type-1 insulin-like growth factor receptor. Nature.

[7] Ward C.W., Hoyne P.A., Flegg R.H. (1995). Insulin and epidermal growth factor receptors contain the cysteine repeat motif found in the tumor necrosis factor receptor. Proteins.

[8] Grassot J., Gouy M., Perriere G., Mouchiroud G. (2006). Origin and molecular evolution of receptor tyrosine kinases with immunoglobulin-like domains. Mol. Biol. Evol..

[9] Windisch J.M., Auer B., Marksteiner R., Lang M.E., Schneider R. (1995). Specific neurotrophin binding to leucine-rich motif peptides of trka and trkb. FEBS Lett..

[10] Reshetnyak A.V., Murray P.B., Shi X., Mo E.S., Mohanty J., Tome F., Bai H., Gunel M., Lax I., Schlessinger J. (2015). Augmentor alpha and beta (fam150) are ligands of the receptor tyrosine kinases alk and ltk: Hierarchy and specificity of ligand-receptor interactions. Proc. Natl. Acad. Sci. USA.

[11] Manni S., Kisko K., Schleier T., Missimer J., Ballmer-Hofer K. (2014). Functional and structural characterization of the kinase insert and the carboxy terminal domain in vegf receptor 2 activation. FASEB J..

[12] Sarabipour S., Hristova K. (2016). Mechanism of fgf receptor dimerization and activation. Nat. Commun..

[13] Jahangiri A., Nguyen A., Chandra A., Sidorov M.K., Yagnik G., Rick J., Han S.W., Chen W., Flanigan P.M., Schneidman-Duhovny D. (2017). Cross-activating c-met/beta1 integrin complex drives metastasis and invasive resistance in cancer. Proc. Natl. Acad. Sci. USA.

[14] Weiss F.U., Daub H., Ullrich A. (1997). Novel mechanisms of rtk signal generation. Curr. Opin. Genet. Dev..

[15] Hubbard S.R., Miller W.T. (2007). Receptor tyrosine kinases: Mechanisms of activation and signaling. Curr. Opin. Cell Biol..

[16] Volinsky N., Kholodenko B.N. (2013). Complexity of receptor tyrosine kinase signal processing. Cold Spring Harbor Perspect. Biol..

[17] Hsieh M.Y., Yang S., Raymond-Stinz M.A., Edwards J.S., Wilson B.S. (2010). Spatio-temporal modeling of signaling protein recruitment to egfr. BMC Syst. Biol..

[18] Ott C.M., Lingappa V.R. (2002). Integral membrane protein biosynthesis: Why topology is hard to predict. J. Cell Sci..

[19] Scharaw S., Iskar M., Ori A., Boncompain G., Laketa V., Poser I., Lundberg E., Perez F., Beck M., Bork P. (2016). The endosomal transcriptional regulator rnf11 integrates degradation and transport of egfr. J. Cell Biol..

[20] Crepaldi T., Pollack A.L., Prat M., Zborek A., Mostov K., Comoglio P.M. (1994). Targeting of the sf/hgf receptor to the basolateral domain of polarized epithelial cells. J. Cell Biol..

[21] Hobert M.E., Kil S.J., Medof M.E., Carlin C.R. (1997). The cytoplasmic juxtamembrane domain of the epidermal growth factor receptor contains a novel autonomous basolateral sorting determinant. J. Biol. Chem..

[22] Mittar S., Ulyatt C., Howell G.J., Bruns A.F., Zachary I., Walker J.H., Ponnambalam S. (2009). Vegfr1 receptor tyrosine kinase localization to the golgi apparatus is calcium-dependent. Exp. Cell Res..

[23] Manickam V., Tiwari A., Jung J.J., Bhattacharya R., Goel A., Mukhopadhyay D., Choudhury A. (2011). Regulation of vascular endothelial growth factor receptor 2 trafficking and angiogenesis by golgi localized t-snare syntaxin 6. Blood.

[24] Tiwari A., Jung J.J., Inamdar S.M., Nihalani D., Choudhury A. (2013). The myosin motor myo1c is required for vegfr2 delivery to the cell surface and for angiogenic signaling. Am. J. Physiol. Heart Circ. Physiol..

[25] Yamada K.H., Nakajima Y., Geyer M., Wary K.K., Ushio-Fukai M., Komarova Y., Malik A.B. (2014). Kif13b regulates angiogenesis through golgi to plasma membrane trafficking of vegfr2. J. Cell Sci..

[26] Chen B., Zhao L., Li X., Ji Y.S., Li N., Xu X.F., Chen Z.Y. (2014). Syntaxin 8 modulates the post-synthetic trafficking of the trka receptor and inflammatory pain transmission. J. Biol. Chem..

[27] Du Y., Shen J., Hsu J.L., Han Z., Hsu M.C., Yang C.C., Kuo H.P., Wang Y.N., Yamaguchi H., Miller S.A. (2014). Syntaxin 6-mediated golgi translocation plays an important role in nuclear functions of egfr through microtubule-dependent trafficking. Oncogene.

[28] Warren C.M., Ziyad S., Briot A., Der A., Iruela-Arispe M.L. (2014). A ligand-independent vegfr2 signaling pathway limits angiogenic responses in diabetes. Sci. Signal..

[29] Maghsoudlou A., Meyer R.D., Rezazadeh K., Arafa E., Pudney J., Hartsough E., Rahimi N. (2016). Rnf121 inhibits angiogenic growth factor signaling by restricting cell surface expression of vegfr-2. Traffic.

[30] Jastrzebski K., Zdzalik-Bielecka D., Maminska A., Kalaidzidis Y., Hellberg C., Miaczynska M. (2017). Multiple routes of endocytic internalization of pdgfrbeta contribute to pdgf-induced stat3 signaling. J. Cell Sci..

[31] Vieira A.V., Lamaze C., Schmid S.L. (1996). Control of egf receptor signaling by clathrin-mediated endocytosis. Science.

[32] Sigismund S., Argenzio E., Tosoni D., Cavallaro E., Polo S., Di Fiore P.P. (2008). Clathrin-mediated internalization is essential for sustained egfr signaling but dispensable for degradation. Dev. Cell.

[33] Tanaka T., Zhou Y., Ozawa T., Okizono R., Banba A., Yamamura T., Oga E., Muraguchi A., Sakurai H. (2018). Ligand-activated epidermal growth factor receptor (egfr) signaling governs endocytic trafficking of unliganded receptor monomers by non-canonical phosphorylation. J. Biol. Chem..

[34] Ewan L.C., Jopling H.M., Jia H., Mittar S., Bagherzadeh A., Howell G.J., Walker J.H., Zachary I.C., Ponnambalam S. (2006). Intrinsic tyrosine kinase activity is required for vascular endothelial growth factor receptor 2 ubiquitination, sorting and degradation in endothelial cells. Traffic.

[35] Lampugnani M.G., Orsenigo F., Gagliani M.C., Tacchetti C., Dejana E. (2006). Vascular endothelial cadherin controls vegfr-2 internalization and signaling from intracellular compartments. J. Cell Biol..

[36] Bruns A.F., Herbert S.P., Odell A.F., Jopling H.M., Hooper N.M., Zachary I.C., Walker J.H., Ponnambalam S. (2010). Ligand-stimulated vegfr2 signaling is regulated by co-ordinated trafficking and proteolysis. Traffic.

[37] Fearnley G.W., Smith G.A., Abdul-Zani I., Yuldasheva N., Mughal N.A., Homer-Vanniasinkam S., Kearney M.T., Zachary I.C., Tomlinson D.C., Harrison M.A. (2016). Vegf-a isoforms program differential vegfr2 signal transduction, trafficking and proteolysis. Biol. Open.

[38] Delos Santos R.C., Bautista S., Lucarelli S., Bone L.N., Dayam R.M., Abousawan J., Botelho R.J., Antonescu C.N. (2017). Selective regulation of clathrin-mediated epidermal growth factor receptor signaling and endocytosis by phospholipase c and calcium. Mol. Biol. Cell.

[39] Bogdanovic E., Coombs N., Dumont D.J. (2009). Oligomerized tie2 localizes to clathrin-coated pits in response to angiopoietin-1. Histochem. Cell Biol..

[40] Wilde A., Beattie E.C., Lem L., Riethof D.A., Liu S.H., Mobley W.C., Soriano P., Brodsky F.M. (1999). Egf receptor signaling stimulates src kinase phosphorylation of clathrin, influencing clathrin redistribution and egf uptake. Cell.

[41] Goh L.K., Huang F., Kim W., Gygi S., Sorkin A. (2010). Multiple mechanisms collectively regulate clathrin-mediated endocytosis of the epidermal growth factor receptor. J. Cell Biol..

[42] Jiang X., Huang F., Marusyk A., Sorkin A. (2003). Grb2 regulates internalization of egf receptors through clathrin-coated pits. Mol. Biol. Cell.

[43] Ioannou M.S., Kulasekaran G., Fotouhi M., Morein J.J., Han C., Tse S., Nossova N., Han T., Mannard E., McPherson P.S. (2017). Intersectin-s interaction with dennd2b facilitates recycling of epidermal growth factor receptor. EMBO Rep..

[44] Puri C., Tosoni D., Comai R., Rabellino A., Segat D., Caneva F., Luzzi P., Di Fiore P.P., Tacchetti C. (2005). Relationships between egfr signaling-competent and endocytosis-competent membrane microdomains. Mol. Biol. Cell.

[45] Rappoport J.Z., Simon S.M. (2009). Endocytic trafficking of activated egfr is ap-2 dependent and occurs through preformed clathrin spots. J. Cell Sci..

[46] Sigismund S., Woelk T., Puri C., Maspero E., Tacchetti C., Transidico P., Di Fiore P.P., Polo S. (2005). Clathrin-independent endocytosis of ubiquitinated cargos. Proc. Natl. Acad. Sci. USA.

[47] Caldieri G., Barbieri E., Nappo G., Raimondi A., Bonora M., Conte A., Verhoef L., Confalonieri S., Malabarba M.G., Bianchi F. (2017). Reticulon 3-dependent er-pm contact sites control egfr nonclathrin endocytosis. Science.

[48] Basagiannis D., Zografou S., Murphy C., Fotsis T., Morbidelli L., Ziche M., Bleck C., Mercer J., Christoforidis S. (2016). Vegf induces signaling and angiogenesis by directing vegfr2 internalisation through macropinocytosis. J. Cell Sci..

[49] Yamabhai M., Anderson R.G. (2002). Second cysteine-rich region of epidermal growth factor receptor contains targeting information for caveolae/rafts. J. Biol. Chem..

[50] Ikeda S., Ushio-Fukai M., Zuo L., Tojo T., Dikalov S., Patrushev N.A., Alexander R.W. (2005). Novel role of arf6 in vascular endothelial growth factor-induced signaling and angiogenesis. Circ. Res..

[51] Yamaguchi T., Lu C., Ida L., Yanagisawa K., Usukura J., Cheng J., Hotta N., Shimada Y., Isomura H., Suzuki M. (2016). Ror1 sustains caveolae and survival signaling as a scaffold of cavin-1 and caveolin-1. Nat. Commun..

[52] Mastick C.C., Brady M.J., Saltiel A.R. (1995). Insulin stimulates the tyrosine phosphorylation of caveolin. J. Cell Biol..

[53] Labrecque L., Royal I., Surprenant D.S., Patterson C., Gingras D., Beliveau R. (2003). Regulation of vascular endothelial growth factor receptor-2 activity by caveolin-1 and plasma membrane cholesterol. Mol. Biol. Cell.

[54] Orlichenko L., Huang B., Krueger E., McNiven M.A. (2006). Epithelial growth factor-induced phosphorylation of caveolin 1 at tyrosine 14 stimulates caveolae formation in epithelial cells. J. Biol. Chem..

[55] Verma N., Keinan O., Selitrennik M., Karn T., Filipits M., Lev S. (2015). Pyk2 sustains endosomal-derived receptor signaling and enhances epithelial-to-mesenchymal transition. Nat. Commun..

[56] Taub N., Teis D., Ebner H.L., Hess M.W., Huber L.A. (2007). Late endosomal traffic of the epidermal growth factor receptor ensures spatial and temporal fidelity of mitogen-activated protein kinase signaling. Mol. Biol. Cell.

[57] Laviolette L.A., Mermoud J., Calvo I.A., Olson N., Boukhali M., Steinlein O.K., Roider E., Sattler E.C., Huang D., Teh B.T. (2017). Negative regulation of egfr signaling by the human folliculin tumour suppressor protein. Nat. Commun..

[58] Kermorgant S., Parker P.J. (2008). Receptor trafficking controls weak signal delivery: A strategy used by c-met for stat3 nuclear accumulation. J. Cell Biol..

[59] Menard L., Parker P.J., Kermorgant S. (2014). Receptor tyrosine kinase c-met controls the cytoskeleton from different endosomes via different pathways. Nat. Commun..

[60] Wang Y., Pennock S.D., Chen X., Kazlauskas A., Wang Z. (2004). Platelet-derived growth factor receptor-mediated signal transduction from endosomes. J. Biol. Chem..

[61] Kofler N., Corti F., Rivera-Molina F., Deng Y., Toomre D., Simons M. (2018). The rab-effector protein rabep2 regulates endosomal trafficking to mediate vascular endothelial growth factor receptor-2 (vegfr2)-dependent signaling. J. Biol. Chem..

[62] Acconcia F., Sigismund S., Polo S. (2009). Ubiquitin in trafficking: The network at work. Exp. Cell Res..

[63] Girnita L., Takahashi S.I., Crudden C., Fukushima T., Worrall C., Furuta H., Yoshihara H., Hakuno F., Girnita A. (2016). Chapter seven-when phosphorylation encounters ubiquitination: A balanced perspective on igf-1r signaling. Prog. Mol. Biol. Transl. Sci..

[64] Smith G.A., Tomlinson D.C., Harrison M.A., Ponnambalam S. (2016). Chapter eight-ubiquitin-mediated regulation of cellular responses to vascular endothelial growth factors. Prog. Mol. Biol. Transl. Sci..

[65] Smith G.A., Fearnley G.W., Abdul-Zani I., Wheatcroft S.B., Tomlinson D.C., Harrison M.A., Ponnambalam S. (2017). Ubiquitination of basal vegfr2 regulates signal transduction and endothelial function. Biol. Open.

[66] Sorkin A., Goh L.K. (2008). Endocytosis and intracellular trafficking of erbbs. Exp. Cell Res..

[67] Gucwa A.L., Brown D.A. (2014). Uim domain-dependent recruitment of the endocytic adaptor protein eps15 to ubiquitin-enriched endosomes. BMC Cell Biol..

[68] Lund P.K., Moats-Staats B.M., Simmons J.G., Hoyt E., D’Ercole A.J., Martin F., Van Wyk J.J. (1985). Nucleotide sequence analysis of a cdna encoding human ubiquitin reveals that ubiquitin is synthesized as a precursor. J. Biol. Chem..

[69] Wiborg O., Pedersen M.S., Wind A., Berglund L.E., Marcker K.A., Vuust J. (1985). The human ubiquitin multigene family: Some genes contain multiple directly repeated ubiquitin coding sequences. EMBO J..

[70] McGrath J.P., Jentsch S., Varshavsky A. (1991). Uba 1: An essential yeast gene encoding ubiquitin-activating enzyme. EMBO J..

[71] Pelzer C., Kassner I., Matentzoglu K., Singh R.K., Wollscheid H.P., Scheffner M., Schmidtke G., Groettrup M. (2007). Ube1l2, a novel e1 enzyme specific for ubiquitin. J. Biol. Chem..

[72] Desterro J.M., Rodriguez M.S., Kemp G.D., Hay R.T. (1999). Identification of the enzyme required for activation of the small ubiquitin-like protein sumo-1. J. Biol. Chem..

[73] Gong L., Yeh E.T. (1999). Identification of the activating and conjugating enzymes of the nedd8 conjugation pathway. J. Biol. Chem..

[74] Van der Veen A.G., Schorpp K., Schlieker C., Buti L., Damon J.R., Spooner E., Ploegh H.L., Jentsch S. (2011). Role of the ubiquitin-like protein urm1 as a noncanonical lysine-directed protein modifier. Proc. Natl. Acad. Sci. USA.

[75] Komatsu M., Chiba T., Tatsumi K., Iemura S., Tanida I., Okazaki N., Ueno T., Kominami E., Natsume T., Tanaka K. (2004). A novel protein-conjugating system for ufm1, a ubiquitin-fold modifier. EMBO J..

[76] Yuan W., Krug R.M. (2001). Influenza b virus ns1 protein inhibits conjugation of the interferon (ifn)-induced ubiquitin-like isg15 protein. EMBO J..

[77] Huang D.T., Miller D.W., Mathew R., Cassell R., Holton J.M., Roussel M.F., Schulman B.A. (2004). A unique e1-e2 interaction required for optimal conjugation of the ubiquitin-like protein nedd8. Nat. Struct. Mol. Biol..

[78] Mizushima N., Noda T., Yoshimori T., Tanaka Y., Ishii T., George M.D., Klionsky D.J., Ohsumi M., Ohsumi Y. (1998). A protein conjugation system essential for autophagy. Nature.

[79] Oweis W., Padala P., Hassouna F., Cohen-Kfir E., Gibbs D.R., Todd E.A., Berndsen C.E., Wiener R. (2016). Trans-binding mechanism of ubiquitin-like protein activation revealed by a uba5-ufm1 complex. Cell Rep..

[80] Mashahreh B., Hassouna F., Soudah N., Cohen-Kfir E., Strulovich R., Haitin Y., Wiener R. (2018). Trans-binding of ufm1 to uba5 stimulates uba5 homodimerization and atp binding. FASEB J..

[81] Cook B.W., Shaw G.S. (2012). Architecture of the catalytic hpn motif is conserved in all e2 conjugating enzymes. Biochem. J..

[82] Wu P.Y., Hanlon M., Eddins M., Tsui C., Rogers R.S., Jensen J.P., Matunis M.J., Weissman A.M., Wolberger C., Pickart C.M. (2003). A conserved catalytic residue in the ubiquitin-conjugating enzyme family. EMBO J..

[83] Morreale F.E., Walden H. (2016). Types of ubiquitin ligases. Cell.

[84] Li W., Bengtson M.H., Ulbrich A., Matsuda A., Reddy V.A., Orth A., Chanda S.K., Batalov S., Joazeiro C.A. (2008). Genome-wide and functional annotation of human e3 ubiquitin ligases identifies mulan, a mitochondrial e3 that regulates the organelle’s dynamics and signaling. PLoS ONE.

[85] Huibregtse J.M., Scheffner M., Beaudenon S., Howley P.M. (1995). A family of proteins structurally and functionally related to the e6-ap ubiquitin-protein ligase. Proc. Natl. Acad. Sci. USA.

[86] Kim H.C., Steffen A.M., Oldham M.L., Chen J., Huibregtse J.M. (2011). Structure and function of a hect domain ubiquitin-binding site. EMBO Rep..

[87] Hatakeyama S., Nakayama K.I. (2003). U-box proteins as a new family of ubiquitin ligases. Biochem. Biophys. Res. Commun..

[88] Kamadurai H.B., Souphron J., Scott D.C., Duda D.M., Miller D.J., Stringer D., Piper R.C., Schulman B.A. (2009). Insights into ubiquitin transfer cascades from a structure of a ubch5b approximately ubiquitin-hect(nedd4l) complex. Mol. Cell.

[89] Deshaies R.J., Joazeiro C.A. (2009). Ring domain e3 ubiquitin ligases. Ann. Rev. Biochem..

[90] Pruneda J.N., Littlefield P.J., Soss S.E., Nordquist K.A., Chazin W.J., Brzovic P.S., Klevit R.E. (2012). Structure of an e3:E2~ub complex reveals an allosteric mechanism shared among ring/u-box ligases. Mol. Cell.

[91] Kirisako T., Kamei K., Murata S., Kato M., Fukumoto H., Kanie M., Sano S., Tokunaga F., Tanaka K., Iwai K. (2006). A ubiquitin ligase complex assembles linear polyubiquitin chains. EMBO J..

[92] Guzzo C.M., Berndsen C.E., Zhu J., Gupta V., Datta A., Greenberg R.A., Wolberger C., Matunis M.J. (2012). Rnf4-dependent hybrid sumo-ubiquitin chains are signals for rap80 and thereby mediate the recruitment of brca1 to sites of DNA damage. Sci. Signal..

[93] Schmidt C.K., Galanty Y., Sczaniecka-Clift M., Coates J., Jhujh S., Demir M., Cornwell M., Beli P., Jackson S.P. (2015). Systematic e2 screening reveals a ube2d-rnf138-ctip axis promoting DNA repair. Nat. Cell Biol..

[94] Han D., Liang J., Lu Y., Xu L., Miao S., Lu L.Y., Song W., Wang L. (2016). Ubiquitylation of rad51d mediated by e3 ligase rnf138 promotes the homologous recombination repair pathway. PLoS ONE.

[95] Uckelmann M., Sixma T.K. (2017). Histone ubiquitination in the DNA damage response. DNA Repair.

[96] Wijnhoven P., Konietzny R., Blackford A.N., Travers J., Kessler B.M., Nishi R., Jackson S.P. (2015). Usp4 auto-deubiquitylation promotes homologous recombination. Mol. Cell.

[97] Beli P., Jackson S.P. (2015). Ubiquitin regulates dissociation of DNA repair factors from chromatin. Oncotarget.

[98] Baranes-Bachar K., Levy-Barda A., Oehler J., Reid D.A., Soria-Bretones I., Voss T.C., Chung D., Park Y., Liu C., Yoon J.B. (2018). The ubiquitin e3/e4 ligase ube4a adjusts protein ubiquitylation and accumulation at sites of DNA damage, facilitating double-strand break repair. Mol. Cell.

[99] Elia A.E., Boardman A.P., Wang D.C., Huttlin E.L., Everley R.A., Dephoure N., Zhou C., Koren I., Gygi S.P., Elledge S.J. (2015). Quantitative proteomic atlas of ubiquitination and acetylation in the DNA damage response. Mol. Cell.

[100] Gatti M., Pinato S., Maiolica A., Rocchio F., Prato M.G., Aebersold R., Penengo L. (2015). Rnf168 promotes noncanonical k27 ubiquitination to signal DNA damage. Cell Rep..

[101] Meyer H.J., Rape M. (2014). Enhanced protein degradation by branched ubiquitin chains. Cell.

[102] Fei C., Li Z., Li C., Chen Y., Chen Z., He X., Mao L., Wang X., Zeng R., Li L. (2013). Smurf1-mediated lys29-linked nonproteolytic polyubiquitination of axin negatively regulates wnt/beta-catenin signaling. Mol. Cell. Biol..

[103] Lu Y., Lee B.H., King R.W., Finley D., Kirschner M.W. (2015). Substrate degradation by the proteasome: A single-molecule kinetic analysis. Science.

[104] Nathan J.A., Kim H.T., Ting L., Gygi S.P., Goldberg A.L. (2013). Why do cellular proteins linked to k63-polyubiquitin chains not associate with proteasomes?. EMBO J..

[105] Liu C.S., Yang-Yen H.F., Suen C.S., Hwang M.J., Yen J.J. (2017). Cbl-mediated k63-linked ubiquitination of jak2 enhances jak2 phosphorylation and signal transduction. Sci. Rep..

[106] Ohtake F., Saeki Y., Ishido S., Kanno J., Tanaka K. (2016). The k48-k63 branched ubiquitin chain regulates nf-kappab signaling. Mol. Cell.

[107] Ohtake F., Tsuchiya H., Saeki Y., Tanaka K. (2018). K63 ubiquitylation triggers proteasomal degradation by seeding branched ubiquitin chains. Proc. Natl. Acad. Sci. USA.

[108] Fiil B.K., Damgaard R.B., Wagner S.A., Keusekotten K., Fritsch M., Bekker-Jensen S., Mailand N., Choudhary C., Komander D., Gyrd-Hansen M. (2013). Otulin restricts met1-linked ubiquitination to control innate immune signaling. Mol. Cell.

[109] Dziedzic S.A., Su Z., Jean Barrett V., Najafov A., Mookhtiar A.K., Amin P., Pan H., Sun L., Zhu H., Ma A. (2018). Abin-1 regulates ripk1 activation by linking met1 ubiquitylation with lys63 deubiquitylation in tnf-rsc. Nat. Cell Biol..

[110] Alkan Z., Duong F.L., Hawkes W.C. (2015). Selenoprotein w controls epidermal growth factor receptor surface expression, activation and degradation via receptor ubiquitination. Biochim. Biophys. Acta.

[111] Haugsten E.M., Zakrzewska M., Brech A., Pust S., Olsnes S., Sandvig K., Wesche J. (2011). Clathrin- and dynamin-independent endocytosis of fgfr3—Implications for signaling. PLoS ONE.

[112] Lanahan A.A., Hermans K., Claes F., Kerley-Hamilton J.S., Zhuang Z.W., Giordano F.J., Carmeliet P., Simons M. (2010). Vegf receptor 2 endocytic trafficking regulates arterial morphogenesis. Dev. Cell.

[113] Lu Z., Je H.S., Young P., Gross J., Lu B., Feng G. (2007). Regulation of synaptic growth and maturation by a synapse-associated e3 ubiquitin ligase at the neuromuscular junction. J. Cell Biol..

[114] Arevalo J.C., Waite J., Rajagopal R., Beyna M., Chen Z.Y., Lee F.S., Chao M.V. (2006). Cell survival through trk neurotrophin receptors is differentially regulated by ubiquitination. Neuron.

[115] Geetha T., Jiang J., Wooten M.W. (2005). Lysine 63 polyubiquitination of the nerve growth factor receptor trka directs internalization and signaling. Mol. Cell.

[116] Geetha T., Wooten M.W. (2008). Trka receptor endolysosomal degradation is both ubiquitin and proteasome dependent. Traffic.

[117] Haglund K., Sigismund S., Polo S., Szymkiewicz I., Di Fiore P.P., Dikic I. (2003). Multiple monoubiquitination of rtks is sufficient for their endocytosis and degradation. Nat. Cell Biol..

[118] Huang F., Zeng X., Kim W., Balasubramani M., Fortian A., Gygi S.P., Yates N.A., Sorkin A. (2013). Lysine 63-linked polyubiquitination is required for egf receptor degradation. Proc. Natl. Acad. Sci. USA.

[119] Hofmann K., Falquet L. (2001). A ubiquitin-interacting motif conserved in components of the proteasomal and lysosomal protein degradation systems. Trends Biochem. Sci..

[120] Polo S., Sigismund S., Faretta M., Guidi M., Capua M.R., Bossi G., Chen H., De Camilli P., Di Fiore P.P. (2002). A single motif responsible for ubiquitin recognition and monoubiquitination in endocytic proteins. Nature.

[121] Barriere H., Nemes C., Lechardeur D., Khan-Mohammad M., Fruh K., Lukacs G.L. (2006). Molecular basis of oligoubiquitin-dependent internalization of membrane proteins in mammalian cells. Traffic.

[122] Baldys A., Raymond J.R. (2009). Critical role of escrt machinery in egfr recycling. Biochemistry.

[123] Li X., Letourneau D., Holleran B., Leduc R., Lavigne P., Lavoie C. (2017). Galphas protein binds ubiquitin to regulate epidermal growth factor receptor endosomal sorting. Proc. Natl. Acad. Sci. USA.

[124] Deshar R., Cho E.B., Yoon S.K., Yoon J.B. (2016). Cc2d1a and cc2d1b regulate degradation and signaling of egfr and tlr4. Biochem. Biophys. Res. Commun..

[125] Soubeyran P., Kowanetz K., Szymkiewicz I., Langdon W.Y., Dikic I. (2002). Cbl-cin85-endophilin complex mediates ligand-induced downregulation of egf receptors. Nature.

[126] Petrelli A., Gilestro G.F., Lanzardo S., Comoglio P.M., Migone N., Giordano S. (2002). The endophilin-cin85-cbl complex mediates ligand-dependent downregulation of c-met. Nature.

[127] Diesenberg K., Beerbaum M., Fink U., Schmieder P., Krauss M. (2015). Sept9 negatively regulates ubiquitin-dependent downregulation of egfr. J. Cell Sci..

[128] Umebayashi K., Stenmark H., Yoshimori T. (2008). Ubc4/5 and c-cbl continue to ubiquitinate egf receptor after internalization to facilitate polyubiquitination and degradation. Mol. Biol. Cell.

[129] Malerod L., Stuffers S., Brech A., Stenmark H. (2007). Vps22/eap30 in escrt-ii mediates endosomal sorting of growth factor and chemokine receptors destined for lysosomal degradation. Traffic.

[130] Longva K.E., Blystad F.D., Stang E., Larsen A.M., Johannessen L.E., Madshus I.H. (2002). Ubiquitination and proteasomal activity is required for transport of the egf receptor to inner membranes of multivesicular bodies. J. Cell Biol..

[131] Chi S., Cao H., Wang Y., McNiven M.A. (2011). Recycling of the epidermal growth factor receptor is mediated by a novel form of the clathrin adaptor protein eps15. J. Biol. Chem..

[132] Jopling H.M., Odell A.F., Pellet-Many C., Latham A.M., Frankel P., Sivaprasadarao A., Walker J.H., Zachary I.C., Ponnambalam S. (2014). Endosome-to-plasma membrane recycling of vegfr2 receptor tyrosine kinase regulates endothelial function and blood vessel formation. Cells.

[133] Hellberg C., Schmees C., Karlsson S., Ahgren A., Heldin C.H. (2009). Activation of protein kinase c alpha is necessary for sorting the pdgf beta-receptor to rab4a-dependent recycling. Mol. Biol. Cell.

[134] Romanelli R.J., LeBeau A.P., Fulmer C.G., Lazzarino D.A., Hochberg A., Wood T.L. (2007). Insulin-like growth factor type-i receptor internalization and recycling mediate the sustained phosphorylation of akt. J. Biol. Chem..

[135] Li X., Lavigne P., Lavoie C. (2015). Gga3 mediates trka endocytic recycling to promote sustained akt phosphorylation and cell survival. Mol. Biol. Cell.

[136] Luiskandl S., Woller B., Schlauf M., Schmid J.A., Herbst R. (2013). Endosomal trafficking of the receptor tyrosine kinase musk proceeds via clathrin-dependent pathways, arf6 and actin. FEBS J..

[137] Mihai C., Chotani M., Elton T.S., Agarwal G. (2009). Mapping of ddr1 distribution and oligomerization on the cell surface by fret microscopy. J. Mol. Biol..

[138] Haugsten E.M., Malecki J., Bjorklund S.M., Olsnes S., Wesche J. (2008). Ubiquitination of fibroblast growth factor receptor 1 is required for its intracellular sorting but not for its endocytosis. Mol. Biol. Cell.

[139] Decker S.J. (1990). Epidermal growth factor and transforming growth factor-alpha induce differential processing of the epidermal growth factor receptor. Biochem. Biophys. Res. Commun..

[140] Stern K.A., Place T.L., Lill N.L. (2008). Egf and amphiregulin differentially regulate cbl recruitment to endosomes and egf receptor fate. Biochem. J..

[141] Baldys A., Gooz M., Morinelli T.A., Lee M.H., Raymond J.R., Luttrell L.M., Raymond J.R. (2009). Essential role of c-cbl in amphiregulin-induced recycling and signaling of the endogenous epidermal growth factor receptor. Biochemistry.

[142] Roepstorff K., Grandal M.V., Henriksen L., Knudsen S.L., Lerdrup M., Grovdal L., Willumsen B.M., van Deurs B. (2009). Differential effects of egfr ligands on endocytic sorting of the receptor. Traffic.

[143] Sehat B., Tofigh A., Lin Y., Trocme E., Liljedahl U., Lagergren J., Larsson O. (2010). Sumoylation mediates the nuclear translocation and signaling of the igf-1 receptor. Sci. Signal..

[144] Knittle A.M., Helkkula M., Johnson M.S., Sundvall M., Elenius K. (2017). Sumoylation regulates nuclear accumulation and signaling activity of the soluble intracellular domain of the erbb4 receptor tyrosine kinase. J. Biol. Chem..

[145] Deng H., Lin Y., Badin M., Vasilcanu D., Stromberg T., Jernberg-Wiklund H., Sehat B., Larsson O. (2011). Over-accumulation of nuclear igf-1 receptor in tumor cells requires elevated expression of the receptor and the sumo-conjugating enzyme ubc9. Biochem. Biophys. Res. Commun..

[146] Visser Smit G.D., Place T.L., Cole S.L., Clausen K.A., Vemuganti S., Zhang G., Koland J.G., Lill N.L. (2009). Cbl controls egfr fate by regulating early endosome fusion. Sci. Signal..

[147] Ravid T., Heidinger J.M., Gee P., Khan E.M., Goldkorn T. (2004). C-cbl-mediated ubiquitinylation is required for epidermal growth factor receptor exit from the early endosomes. J. Biol. Chem..

[148] Severe N., Miraoui H., Marie P.J. (2011). The casitas b lineage lymphoma (cbl) mutant g306e enhances osteogenic differentiation in human mesenchymal stromal cells in part by decreased cbl-mediated platelet-derived growth factor receptor alpha and fibroblast growth factor receptor 2 ubiquitination. J. Biol. Chem..

[149] Reddi A.L., Ying G., Duan L., Chen G., Dimri M., Douillard P., Druker B.J., Naramura M., Band V., Band H. (2007). Binding of cbl to a phospholipase cgamma1-docking site on platelet-derived growth factor receptor beta provides a dual mechanism of negative regulation. J. Biol. Chem..

[150] Rorsman C., Tsioumpekou M., Heldin C.H., Lennartsson J. (2016). The ubiquitin ligases c-cbl and cbl-b negatively regulate platelet-derived growth factor (pdgf) bb-induced chemotaxis by affecting pdgf receptor beta (pdgfrbeta) internalization and signaling. J. Biol. Chem..

[151] Takahashi Y., Shimokawa N., Esmaeili-Mahani S., Morita A., Masuda H., Iwasaki T., Tamura J., Haglund K., Koibuchi N. (2011). Ligand-induced downregulation of trka is partly regulated through ubiquitination by cbl. FEBS Lett..

[152] Hyndman B.D., Crupi M.J.F., Peng S., Bone L.N., Rekab A.N., Lian E.Y., Wagner S.M., Antonescu C.N., Mulligan L.M. (2017). Differential recruitment of e3 ubiquitin ligase complexes regulates ret isoform internalization. J. Cell Sci..

[153] Sharfe N., Freywald A., Toro A., Roifman C.M. (2003). Ephrin-a1 induces c-cbl phosphorylation and epha receptor down-regulation in t cells. J. Immunol..

[154] Fasen K., Cerretti D.P., Huynh-Do U. (2008). Ligand binding induces cbl-dependent ephb1 receptor degradation through the lysosomal pathway. Traffic.

[155] Duval M., Bedard-Goulet S., Delisle C., Gratton J.P. (2003). Vascular endothelial growth factor-dependent down-regulation of flk-1/kdr involves cbl-mediated ubiquitination. Consequences on nitric oxide production from endothelial cells. J. Biol. Chem..

[156] Kobayashi S., Sawano A., Nojima Y., Shibuya M., Maru Y. (2004). The c-cbl/cd2ap complex regulates vegf-induced endocytosis and degradation of flt-1 (vegfr-1). FASEB J..

[157] Parks E.E., Ceresa B.P. (2014). Cell surface epidermal growth factor receptors increase src and c-cbl activity and receptor ubiquitylation. J. Biol. Chem..

[158] Emdal K.B., Pedersen A.K., Bekker-Jensen D.B., Tsafou K.P., Horn H., Lindner S., Schulte J.H., Eggert A., Jensen L.J., Francavilla C. (2015). Temporal proteomics of ngf-trka signaling identifies an inhibitory role for the e3 ligase cbl-b in neuroblastoma cell differentiation. Sci. Signal..

[159] Sirisaengtaksin N., Gireud M., Yan Q., Kubota Y., Meza D., Waymire J.C., Zage P.E., Bean A.J. (2014). Ube4b protein couples ubiquitination and sorting machineries to enable epidermal growth factor receptor (egfr) degradation. J. Biol. Chem..

[160] Gschweitl M., Ulbricht A., Barnes C.A., Enchev R.I., Stoffel-Studer I., Meyer-Schaller N., Huotari J., Yamauchi Y., Greber U.F., Helenius A. (2016). A spopl/cullin-3 ubiquitin ligase complex regulates endocytic trafficking by targeting eps15 at endosomes. eLife.

[161] Song R., Peng W., Zhang Y., Lv F., Wu H.K., Guo J., Cao Y., Pi Y., Zhang X., Jin L. (2013). Central role of e3 ubiquitin ligase mg53 in insulin resistance and metabolic disorders. Nature.

[162] Nagarajan A., Petersen M.C., Nasiri A.R., Butrico G., Fung A., Ruan H.B., Kursawe R., Caprio S., Thibodeau J., Bourgeois-Daigneault M.C. (2016). March1 regulates insulin sensitivity by controlling cell surface insulin receptor levels. Nat. Commun..

[163] Sehat B., Andersson S., Girnita L., Larsson O. (2008). Identification of c-cbl as a new ligase for insulin-like growth factor-i receptor with distinct roles from mdm2 in receptor ubiquitination and endocytosis. Cancer Res..

[164] Vecchione A., Marchese A., Henry P., Rotin D., Morrione A. (2003). The grb10/nedd4 complex regulates ligand-induced ubiquitination and stability of the insulin-like growth factor i receptor. Mol. Cell. Biol..

[165] Girnita L., Girnita A., Larsson O. (2003). Mdm2-dependent ubiquitination and degradation of the insulin-like growth factor 1 receptor. Proc. Natl. Acad. Sci. USA.

[166] Monami G., Emiliozzi V., Morrione A. (2008). Grb10/nedd4-mediated multiubiquitination of the insulin-like growth factor receptor regulates receptor internalization. J. Cell. Physiol..

[167] Murdaca J., Treins C., Monthouel-Kartmann M.N., Pontier-Bres R., Kumar S., Van Obberghen E., Giorgetti-Peraldi S. (2004). Grb10 prevents nedd4-mediated vascular endothelial growth factor receptor-2 degradation. J. Biol. Chem..

[168] Hasseine L.K., Murdaca J., Suavet F., Longnus S., Giorgetti-Peraldi S., Van Obberghen E. (2007). Hrs is a positive regulator of vegf and insulin signaling. Exp. Cell Res..

[169] Ye Y., Scheel H., Hofmann K., Komander D. (2009). Dissection of usp catalytic domains reveals five common insertion points. Mol. Biosyst..

[170] Mevissen T.E., Hospenthal M.K., Geurink P.P., Elliott P.R., Akutsu M., Arnaudo N., Ekkebus R., Kulathu Y., Wauer T., El Oualid F. (2013). Otu deubiquitinases reveal mechanisms of linkage specificity and enable ubiquitin chain restriction analysis. Cell.

[171] Seki T., Gong L., Williams A.J., Sakai N., Todi S.V., Paulson H.L. (2013). Josd1, a membrane-targeted deubiquitinating enzyme, is activated by ubiquitination and regulates membrane dynamics, cell motility, and endocytosis. J. Biol. Chem..

[172] Abdul Rehman S.A., Kristariyanto Y.A., Choi S.Y., Nkosi P.J., Weidlich S., Labib K., Hofmann K., Kulathu Y. (2016). Mindy-1 is a member of an evolutionarily conserved and structurally distinct new family of deubiquitinating enzymes. Mol. Cell.

[173] Kapoor N., Niu J., Saad Y., Kumar S., Sirakova T., Becerra E., Li X., Kolattukudy P.E. (2015). Transcription factors stat6 and klf4 implement macrophage polarization via the dual catalytic powers of mcpip. J. Immunol..

[174] Liang J., Saad Y., Lei T., Wang J., Qi D., Yang Q., Kolattukudy P.E., Fu M. (2010). Mcp-induced protein 1 deubiquitinates traf proteins and negatively regulates jnk and nf-kappab signaling. J. Exp. Med..

[175] Komander D., Clague M.J., Urbe S. (2009). Breaking the chains: Structure and function of the deubiquitinases. Nat. Rev. Mol. Cell Biol..

[176] Grou C.P., Pinto M.P., Mendes A.V., Domingues P., Azevedo J.E. (2015). The de novo synthesis of ubiquitin: Identification of deubiquitinases acting on ubiquitin precursors. Sci. Rep..

[177] Komander D., Lord C.J., Scheel H., Swift S., Hofmann K., Ashworth A., Barford D. (2008). The structure of the cyld usp domain explains its specificity for lys63-linked polyubiquitin and reveals a b box module. Mol. Cell.

[178] Kristariyanto Y.A., Abdul Rehman S.A., Weidlich S., Knebel A., Kulathu Y. (2017). A single miu motif of mindy-1 recognizes k48-linked polyubiquitin chains. EMBO Rep..

[179] Sato Y., Okatsu K., Saeki Y., Yamano K., Matsuda N., Kaiho A., Yamagata A., Goto-Ito S., Ishikawa M., Hashimoto Y. (2017). Structural basis for specific cleavage of lys6-linked polyubiquitin chains by usp30. Nat. Struct. Mol. Biol..

[180] Peth A., Uchiki T., Goldberg A.L. (2010). Atp-dependent steps in the binding of ubiquitin conjugates to the 26s proteasome that commit to degradation. Mol. Cell.

[181] Harrigan J.A., Jacq X., Martin N.M., Jackson S.P. (2018). Deubiquitylating enzymes and drug discovery: Emerging opportunities. Nat. Rev. Drug Discov..

[182] Duex J.E., Sorkin A. (2009). Rna interference screen identifies usp18 as a regulator of epidermal growth factor receptor synthesis. Mol. Biol. Cell.

[183] Duex J.E., Comeau L., Sorkin A., Purow B., Kefas B. (2011). Usp18 regulates epidermal growth factor (egf) receptor expression and cancer cell survival via microrna-7. J. Biol. Chem..

[184] McCullough J., Clague M.J., Urbe S. (2004). Amsh is an endosome-associated ubiquitin isopeptidase. J. Cell Biol..

[185] McCullough J., Row P.E., Lorenzo O., Doherty M., Beynon R., Clague M.J., Urbe S. (2006). Activation of the endosome-associated ubiquitin isopeptidase amsh by stam, a component of the multivesicular body-sorting machinery. Curr. Biol. CB.

[186] Savio M.G., Wollscheid N., Cavallaro E., Algisi V., Di Fiore P.P., Sigismund S., Maspero E., Polo S. (2016). Usp9x controls egfr fate by deubiquitinating the endocytic adaptor eps15. Curr. Biol. CB.

[187] Guo Y.Y., Lu Y., Zheng Y., Chen X.R., Dong J.L., Yuan R.R., Huang S.H., Yu H., Wang Y., Chen Z.Y. (2017). Ubiquitin c-terminal hydrolase l1 (uch-l1) promotes hippocampus-dependent memory via its deubiquitinating effect on trkb. J. Neurosci..

[188] Mizuno E., Kobayashi K., Yamamoto A., Kitamura N., Komada M. (2006). A deubiquitinating enzyme ubpy regulates the level of protein ubiquitination on endosomes. Traffic.

[189] Niendorf S., Oksche A., Kisser A., Lohler J., Prinz M., Schorle H., Feller S., Lewitzky M., Horak I., Knobeloch K.P. (2007). Essential role of ubiquitin-specific protease 8 for receptor tyrosine kinase stability and endocytic trafficking in vivo. Mol. Cell. Biol..

[190] Oh Y.M., Lee S.B., Choi J., Suh H.Y., Shim S., Song Y.J., Kim B., Lee J.M., Oh S.J., Jeong Y. (2014). Usp8 modulates ubiquitination of lrig1 for met degradation. Sci. Rep..

[191] Berlin I., Schwartz H., Nash P.D. (2010). Regulation of epidermal growth factor receptor ubiquitination and trafficking by the usp8.Stam complex. J. Biol. Chem..

[192] Reincke M., Sbiera S., Hayakawa A., Theodoropoulou M., Osswald A., Beuschlein F., Meitinger T., Mizuno-Yamasaki E., Kawaguchi K., Saeki Y. (2015). Mutations in the deubiquitinase gene usp8 cause cushing's disease. Nat. Genet..

[193] Smith G.A., Fearnley G.W., Abdul-Zani I., Wheatcroft S.B., Tomlinson D.C., Harrison M.A., Ponnambalam S. (2016). Vegfr2 trafficking, signaling and proteolysis is regulated by the ubiquitin isopeptidase usp8. Traffic.

[194] Jian F., Cao Y., Bian L., Sun Q. (2015). Usp8: A novel therapeutic target for cushing's disease. Endocrine.

[195] Byun S., Lee S.Y., Lee J., Jeong C.H., Farrand L., Lim S., Reddy K., Kim J.Y., Lee M.H., Lee H.J. (2013). Usp8 is a novel target for overcoming gefitinib resistance in lung cancer. Clin. Cancer Res..

